# Allele-Specific Thresholds of Eluted Ligands for T-Cell Epitope Prediction

**DOI:** 10.1016/j.mcpro.2021.100122

**Published:** 2021-07-22

**Authors:** Brian Reardon, Zeynep Koşaloğlu-Yalçın, Sinu Paul, Bjoern Peters, Alessandro Sette

**Affiliations:** 1 Division of Vaccine Discovery, La Jolla Institute for Immunology, La Jolla, California, USA; 2 Department of Medicine, University of California, San Diego, San Diego, California, USA

**Keywords:** HLA-class I, T-cell epitope, eluted ligand, binding threshold, MHC repertoire, BA, binding affinity, EL, eluted ligand, FN, false negative, FP, false positive, HLA, human leukocyte antigen, IEDB, Immune Epitope Database, NPV, negative predictive value, PPV, positive predictive value, ROC, receiver operating characteristic, TN, true negative, TP, true positive

## Abstract

A common strategy for predicting candidate human leukocyte antigen class I T-cell epitopes is to use an affinity-based threshold of 500 nM. Although a 500 nM threshold across alleles effectively identifies most epitopes across alleles, findings showing that major histocompatibility complex repertoire sizes vary by allele indicate that using thresholds specific to individual alleles may improve epitope identification. In this work, we compare different strategies utilizing common and allele-specific thresholds to identify an optimal approach for T-cell epitope prediction. First, we confirmed previous observations that different human leukocyte antigen class I alleles correspond with varying repertoire sizes. Here, we define general and allele-specific thresholds that capture 80% of eluted ligands and a different set of thresholds associated with capturing 9-mer T-cell epitopes at 80% sensitivity. Our analysis revealed that allele-specific threshold performance was roughly equivalent to that of a common threshold when considering a relatively large number of alleles. However, when predicting epitopes for only a few alleles, the use of allele-specific thresholds would be preferable. Finally, we present here for public use a set of allele-specific thresholds that may be used to identify T-cell epitopes at 80% sensitivity.

## Highlights

• Confirmed findings that different HLA class I alleles have varying repertoire sizes.
• Defined common and allele-specific thresholds that capture 80% of eluted ligands.
• Defined common and allele-specific thresholds that capture 80% of T-cell epitopes.
• Allele-specific thresholds perform more consistently when analyzing a few alleles.

## Graphical Abstract

**Figure:**
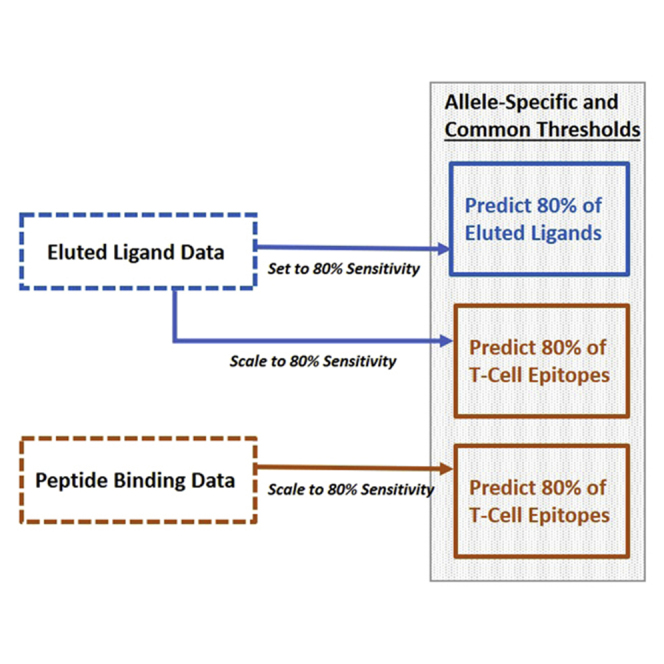

## In Brief

Candidate HLA class I T-cell epitopes are frequently identified using a single threshold across multiple alleles. However, previous studies have demonstrated that different HLA class I alleles have varying repertoire sizes. In this study, we investigated the performance of different strategies to perform epitope-binding prediction for multiple HLA class I alleles simultaneously. We identified and present here a set of general and allele-specific thresholds that may be used to identify eluted ligands and T-cell epitopes at 80% sensitivity.

Computational prediction tools are commonly used to identify epitopes that are presented on major histocompatibility complex (MHC) class I molecules to CD8+ T cells. Most MHC-binding prediction tools are based on machine learning algorithms trained on datasets of known peptide–MHC binding affinities and can accurately predict the binding capacity of a peptide to a given MHC. IC_50_ values are usually utilized to measure the binding affinity (BA): IC_50_ values are defined as the concentration that inhibits 50% binding of a labeled reference peptide, and low IC_50_ values correspond to high binding affinities.

More recently, algorithms are also being trained on ligand elution data. As eluted ligands (ELs) passed through the natural antigen processing and presentation pathway, ligand elution data inherently contain information that is not available when only peptide–MHC binding is considered (1). Also, as high-throughput ligand elution assays allow identifying thousands of natural ligands with a single experiment, large training datasets have become available (2, 3). These methods usually provide a score to estimate a peptide's likelihood of being eluted from a given human leukocyte antigen (HLA) molecule and have been shown to perform better in predicting epitopes than methods that are solely based on binding-affinity data (4, 5).

In this study, we used the state-of-the-art prediction tool NetMHCpan 4.0, which was trained with both BA and EL data (4). Using such prediction tools, different strategies are available to select peptides as potential T-cell epitope candidates. One approach is to use set thresholds for the predicted IC_50_ values or EL scores. Alternatively, percentile ranks that describe the percentile of the prediction among a large number of random peptides can be utilized.

In a most commonly encountered practical application of epitope predictions, predictions for multiple alleles are performed simultaneously to accommodate HLA polymorphisms commonly present in the study population of interest. However, we previously reported that different HLA class I alleles have different repertoire sizes (6). Accordingly, here, we wanted to explore whether using allele-specific thresholds would perform better than a common “one-size-fits-all” threshold.

In this study, we first compared the performance of BA and EL predictions in identifying ELs. As expected, we found that percentile ranks of ligand elution predictions (EL Rank) are superior to BA predictions in this context. Next, we established common and allele-specific thresholds that predict ELs with 80% sensitivity. Using these newly established thresholds, we then evaluated the findings from our previous study about different HLA class I alleles having varying repertoire sizes (6) and found that the repertoire sizes described in Paul *et al.* in 2013 are highly correlated to those found in the present study. We further compared the established common and allele-specific thresholds that were derived from ELs to predict ELs and analyzed their performance in predicting ELs and T-cell epitopes. We found that these thresholds are not suitable to predict T-cell epitopes and established additional thresholds that were also derived from ELs but scaled to predict 80% of epitopes. Our analysis revealed that either common or allele-specific thresholds necessary to capture 80% of reported ELs were significantly more stringent than those required to predict 80% of known T-cell epitopes. Our analysis further revealed that allele-specific threshold performance was roughly equivalent to that of a common threshold when considering a relatively large number of alleles. However, when predicting epitopes for only a few alleles, the use of allele-specific thresholds would be preferable.

## Experimental Procedures

### Selection of Eluted 9-Mer Ligands From Defined HLA Class I Alleles

**Fig. 1:**
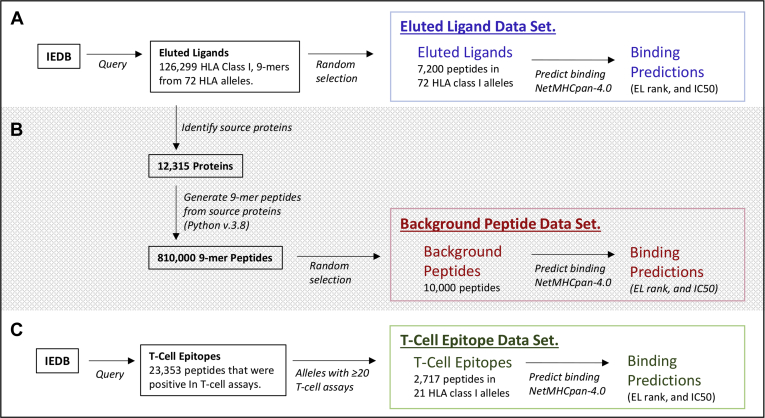
**Generation of peptide datasets used for binding threshold analyses.***A*, the eluted ligand dataset is composed of 100 9-mer peptides for each of 72 HLA class I alleles and binding predictions for each peptide to its corresponding HLA allele. *B*, the background peptide dataset is composed of 10,000 9-mers and binding predictions for each of 72 HLA class I alleles. *C*, the T-cell epitope dataset was selected from an initial set of ~23,000 peptides. Only epitopes from alleles having 20 or more T-cell assays were retained for inclusion in this dataset composed of 2492 9-mers and binding predictions for each peptide to its corresponding HLA allele. HLA, human leukocyte antigen.

We selected human HLA class I ELs by querying the Immune Epitope Database (IEDB) (7, 8, 9) on 1/29/2020 using the following parameters; MHC ligand assays: MHC ligand elution assay, positive assays only, no B-cell assays, no T-cell assays, MHC restriction class I; host: *Homo sapiens*; epitope: any epitopes; disease: any disease. The search results with Internationalized Resource Identifiers were exported to a comma-separated file. The resulting download had 763,213 records. All records from PubMed IDs 28188227 and 29393594 were excluded from analysis because the ligands were primarily eluted from transgenic rat T cells. Further filtering to retain only a single instance of 9-mer ligands with defined alleles resulted in 126,299 remaining ELs. Alleles having at least 100 ligands were selected, resulting in 123,707 ligands from 72 HLA alleles (Table 1). The number of ligands considered ranged from 101 to over 8000. From these ligands, 100 ligands per allele were randomly selected. These ligands comprised the positive data points in the eluted ligand dataset (Fig. 1).

**Table 1:** Unique eluted ligands identified from 72 HLA class I alleles selected for threshold analysis

Allele	Ligands	Allele	Ligands	Allele	Ligands
HLA-A∗01:01	2217	HLA-B∗18:03	105	HLA-B∗51:01	2034
HLA-A∗02:01	8041	HLA-B∗27:01	1877	HLA-B∗51:08	486
HLA-A∗02:03	1212	HLA-B∗27:02	1259	HLA-B∗54:01	608
HLA-A∗02:04	2306	HLA-B∗27:03	468	HLA-B∗56:01	399
HLA-A∗02:05	211	HLA-B∗27:04	761	HLA-B∗57:01	5707
HLA-A∗02:07	2974	HLA-B∗27:05	6738	HLA-B∗57:03	2266
HLA-A∗03:01	2227	HLA-B∗27:06	747	HLA-B∗58:01	1943
HLA-A∗11:01	1445	HLA-B∗27:07	1087	HLA-B∗73:01	104
HLA-A∗23:01	101	HLA-B∗27:08	938	HLA-C∗01:02	1163
HLA-A∗24:02	3290	HLA-B∗27:09	1004	HLA-C∗02:02	1533
HLA-A∗24:06	115	HLA-B∗35:01	1032	HLA-C∗03:03	883
HLA-A∗24:13	148	HLA-B∗35:03	128	HLA-C∗03:04	1661
HLA-A∗25:01	916	HLA-B∗35:08	107	HLA-C∗04:01	1285
HLA-A∗29:02	5101	HLA-B∗39:01	584	HLA-C∗05:01	2559
HLA-A∗30:01	594	HLA-B∗39:24	129	HLA-C∗06:02	1587
HLA-A∗31:01	763	HLA-B∗40:01	989	HLA-C∗07:01	323
HLA-A∗32:01	418	HLA-B∗40:02	7846	HLA-C∗07:02	789
HLA-A∗68:02	1169	HLA-B∗41:01	329	HLA-C∗08:02	2232
HLA-B∗07:02	5634	HLA-B∗44:02	2119	HLA-C∗12:02	6819
HLA-B∗08:01	2984	HLA-B∗44:03	2556	HLA-C∗12:03	1542
HLA-B∗14:02	229	HLA-B∗45:01	513	HLA-C∗14:02	1609
HLA-B∗15:01	4111	HLA-B∗46:01	1341	HLA-C∗15:02	1639
HLA-B∗15:02	1590	HLA-B∗49:01	158	HLA-C∗16:01	2032
HLA-B∗18:01	1048	HLA-B∗50:01	427	HLA-C∗17:01	418

### Assembly of HLA Class I Restricted 9-Mer Epitopes

To generate a set of T-cell epitopes suitable for analysis, we queried the IEDB website on 11/15/2019 using the following parameters; positive assays only, no B-cell assays, no MHC ligand assays, MHC restriction class I; host: *Homo sapiens*. This query resulted in a download of 23,353 records. The categories of positive assays considered in order of ‘positivity’ were tetramer > positive-high > positive > positive-intermediate > positive > positive-low. The inclusion of positive tetramers here defines the most stringent level of MHC restriction. We retained only 9-mer epitopes associated with a defined HLA restriction (specified allele group and specific HLA protein). For epitopes having more than one assay result for a given allele (*e.g.*, positive-high and positive-intermediate for epitope 'X'/HLA allele 'Y'), we only retained the most positive epitope.

Similarly, we retained a single copy of each epitope sequence. Epitopes having identical sequences and MHC alleles but located in multiple proteins or occurring more than once in a single protein were considered as a single instance (retaining the most positive result). Finally, we only included data for a set of 21 HLA class I alleles having at least 20 total assay results across all levels of positivity (Fig. 1, Table 2).

**Table 2:** Unique T-cell epitopes identified from 21 HLA class I alleles selected for threshold analysis

Allele	Total epitopes (per allele)
HLA-A∗02:01	1465
HLA-A∗24:02	155
HLA-B∗07:02	143
HLA-B∗35:01	152
HLA-A∗11:01	96
HLA-B∗08:01	119
HLA-A∗03:01	73
HLA-A∗01:01	68
HLA-B∗15:01	57
HLA-B∗57:01	44
HLA-B∗40:01	37
HLA-B∗58:01	42
HLA-B∗27:05	50
HLA-A∗02:06	32
HLA-B∗51:01	25
HLA-C∗06:02	36
HLA-B∗53:01	24
HLA-A∗29:02	26
HLA-A∗02:03	26
HLA-A∗02:02	25
HLA-A∗68:02	22
Total epitopes (nonredundant)	2492

A total of 2717 epitopes from 21 HLA class I alleles having ≥20 total positive assays were retained for threshold analysis.

### Generating Background Peptides to Analyze Binding Thresholds in 9-Mer Peptides

A 'control' background dataset composed of 10,000 peptides was assembled using custom Python scripts. We identified 12,315 source proteins from which the positive peptides of Table 1 were derived. We then generated over 800,000 9-mer peptide sequences from these source proteins and randomly selected a subset of 10,000 peptides. By using all available source proteins and randomly selecting peptides, we avoided introducing any bias for source proteins. These 10,000 peptides were used as background control peptides for each tested allele by assigning them the same HLA restriction. If peptide sequences were present in both the random background and positive datasets, we only retained it in the positive ligand dataset. These randomly generated 10,000 background peptides assigned to the analyzed 72 alleles comprised the negative data points in the eluted ligand dataset (Fig. 1).

### Prediction of Binding Affinities and EL Ranks

We determined the predicted binding capacities for all positives and negatives in the EL and T-cell epitope datasets. We utilized the MHC binding prediction tool NetMHCpan 4.0 as implemented on the IEDB Analysis Resource (4, 10). Specifically, we used the NetMHCpan 4.0 BA method to calculate the predicted BA (IC_50_) and corresponding percentile ranks (BA rank), and we used the NetMHCpan 4.0 EL method to generate EL scores and corresponding percentile ranks (EL rank). Of note, only about 30% of the 72,000 ligand–HLA pairs included in our study were also included in the training of NetMHCpan 4.0. Distributions of predicted IC_50_ and EL ranks of ligands and background peptides are displayed in supplemental Figs. S1 and S2 and highlight that ligands of each allele are predicted to bind significantly stronger than the background peptides.

### Assessment of HLA Class I Binding Threshold Performance

Performance of different IC_50_ and EL rank thresholds was determined using Python, version 3.8, with the 'pandas' and 'sklearn' packages. When analyzing a given threshold, we considered positive peptides that were predicted below the threshold as true positives (TPs) and positive peptides predicted above the threshold as false negatives (FNs). We considered negative peptides that were predicted below the threshold of interest as false positives (FPs) and negative peptides predicted above the threshold as true negatives (TNs).

We then used these counts to calculate various measures of performance:

- Sensitivity: TP/(TP + FN)
- Specificity: TN/(TN + FP)
- Positive predictive value (PPV): TP/(TP + FP)
- Negative predictive value (NPV): TN/(TN + FN)
- Accuracy: (TP + TN)/(TP + TN + FP + FN)

We additionally assessed performance by receiver operating characteristic (ROC) analyses measuring the area under the ROC curve.

### Experimental Design and Statistical Rationale

In querying the IEDB for HLA class I T-cell epitopes, we selected epitopes at any level of 'positivity'. To compare our datasets of 100 ELs per allele, we generated a background dataset comprised of 10,000 peptides. The control background dataset thus corresponded to an approximate 1:100 ratio of positives to controls for ELs. For threshold analysis in T-cell epitope datasets, this corresponded to an approximate 1:200 ratio of positives to background controls. We generated an independent set of T-cell epitopes from a subset of six alleles to validate results from this analysis.

## Results

### Comparison of IC_50_ and EL Rank Prediction Performance in a Multiallele Setting

**Fig. 2:**
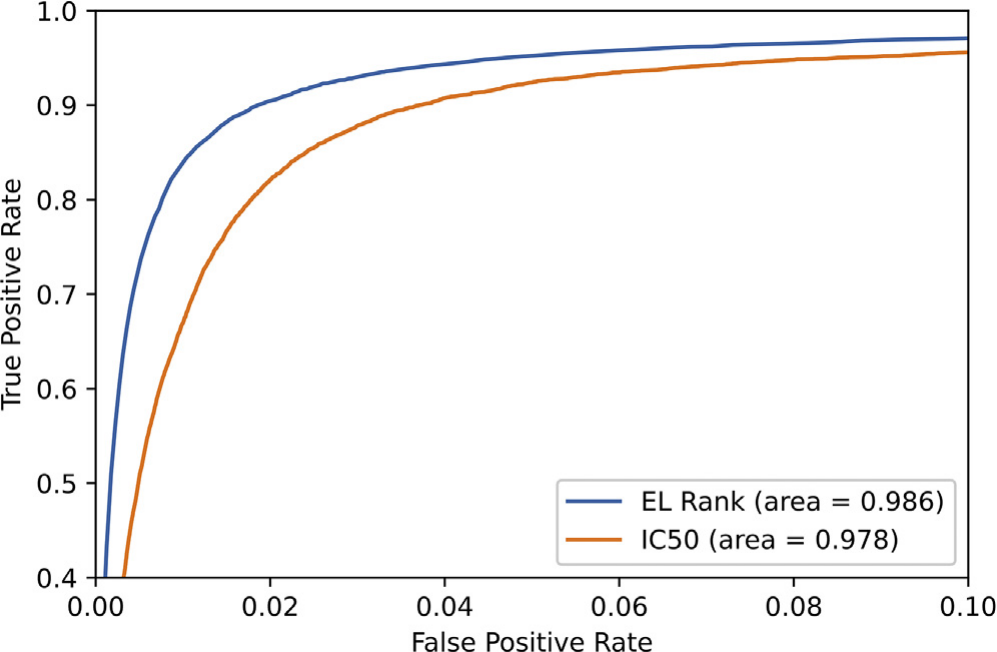
**EL rank predictions outperform** IC_50_**predictions in identifying HLA class I eluted ligands.** EL rank and IC_50_ thresholds were tested using the eluted ligand dataset. Receiver operating characteristics (ROC) curves and area under the ROC curve (AUC) are displayed for EL rank (*blue*) and IC_50_ (*orange*). EL, eluted ligand; HLA, human leukocyte antigen.

First, we addressed the issue of whether, when predictions for various alleles are performed simultaneously, it is preferable to use a common IC_50_ or EL rank threshold to maximize epitope prediction efficiency. We used our dataset of human ELs and performed ROC analyses to compare the performance of the two prediction outputs, EL rank, and IC_50_, in discriminating ELs (positives) from background peptides (negatives). The results of this analysis showed a higher area under the ROC curve using EL rank compared with IC_50_-based binding prediction scores, confirming previous reports (4, 5) (Fig. 2).

### Predicted Binding Affinities and EL Ranks of ELs Vary as a Function of Different HLA Class I Alleles

**Supplemental_Figure-3:**
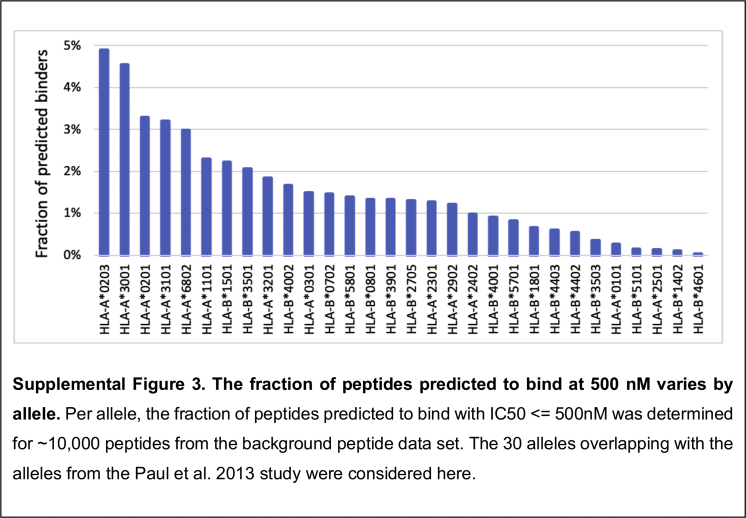


**Supplemental_Figure-4:**
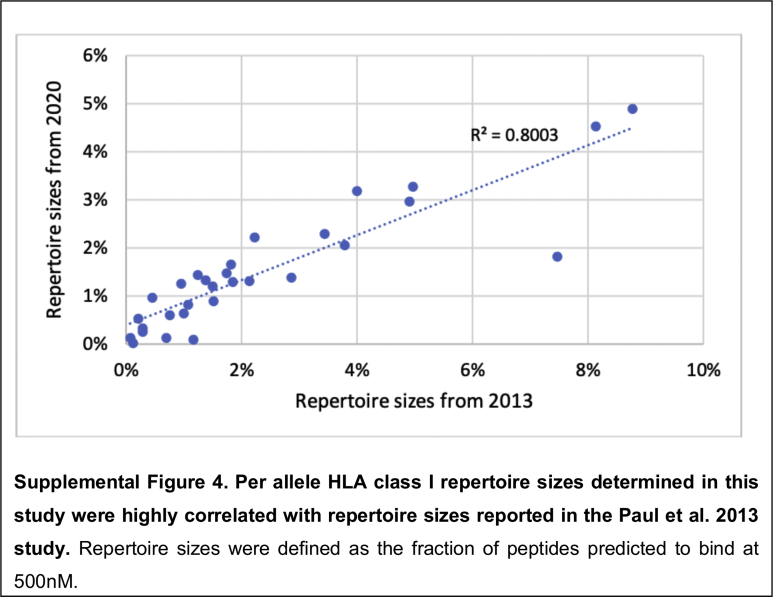


We had previously reported that different HLA class I alleles have different repertoire sizes (6). To validate those results, we performed NetMHCpan 4.0 predictions using the background control peptides described above and calculated the fraction of peptides predicted to bind at 500 nM. As expected, the results confirmed that the different alleles are associated with varying repertoire sizes (6) (supplemental Fig. S3). The repertoire sizes observed in the Paul *et al.* (2013) report (6) and in the current analysis were strongly correlated (supplemental Fig. S4).

**Fig. 3:**
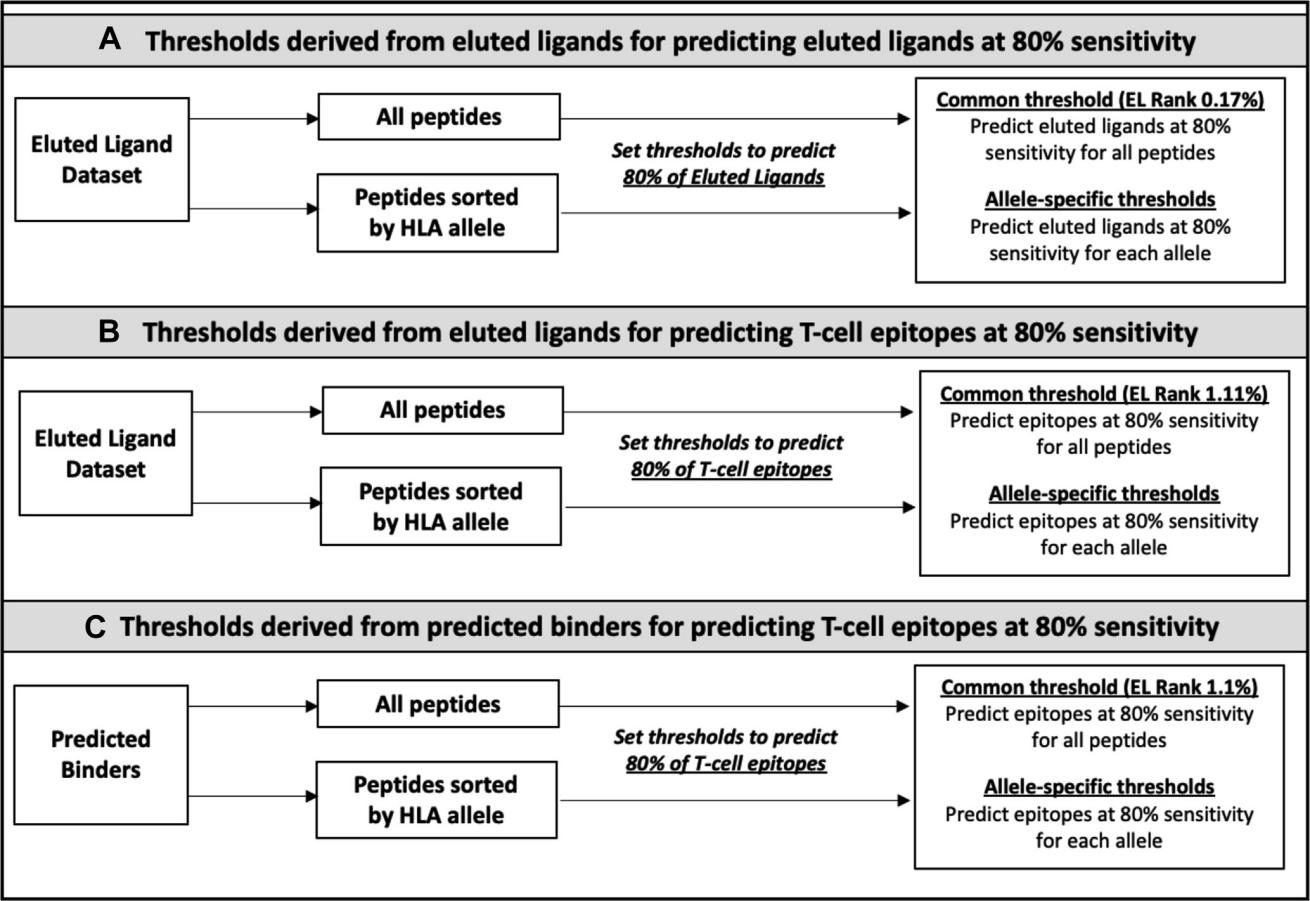
**Generating thresholds for predicting eluted ligand and T-cell epitope.***A*, thresholds derived from eluted ligands (ELs) for predicting ELs at 80% sensitivity. 7200 ELs with corresponding EL rank–based binding predictions were sorted into 72 allele-specific peptide lists or retained in a single list composed of all alleles combined. Allele-specific thresholds were determined by setting the sensitivity to 80% for each allele. Common thresholds were determined by setting the sensitivity to 80% for all alleles combined. *B*, thresholds derived from ELs for predicting T-cell epitopes at 80% sensitivity. The sensitivity of EL rank thresholds was first measured in the T-cell epitope dataset. To generate thresholds for predicting T-cell epitopes, allele-specific and common EL rank thresholds were increased until an average sensitivity of 80% was reached across alleles. *C*, thresholds derived from predicted binders for predicting T-cell epitopes at 80% sensitivity. Using the background peptides, for each allele, we determined the proportion of peptides that bind at 500 nM. These proportion values were then used as thresholds, and the sensitivity of predicting T-cell epitopes was measured. To generate thresholds for predicting T-cell epitopes, allele-specific and common EL rank thresholds were increased until an average sensitivity of 80% was reached across alleles.

**Fig. 4:**
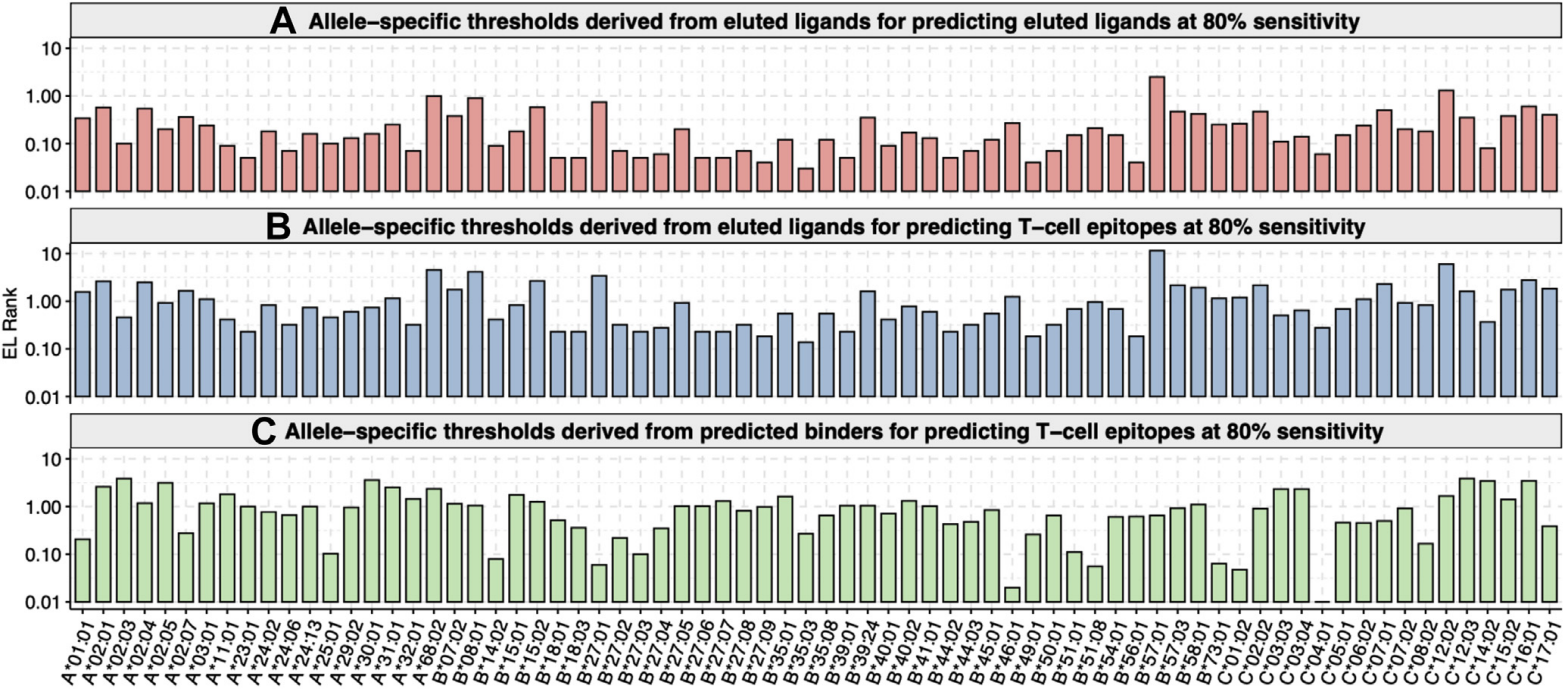
**Allele-specific EL rank thresholds for 72 alleles.** Thresholds were derived from eluted ligands (ELs) for (*A*) predicting ELs at 80% sensitivity and (*B*) predicting T-cell epitopes at 80% sensitivity. *C*, thresholds were derived from a set of predicted binders to predict T-cell epitopes at 80% sensitivity.

**Supplemental_Figure-5:**
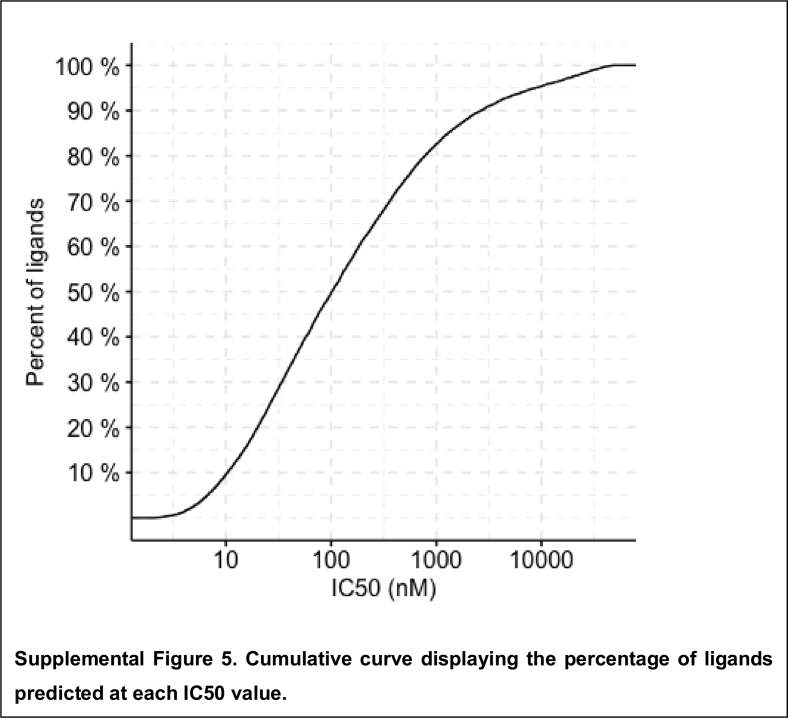


**Supplemental_Figure-6:**
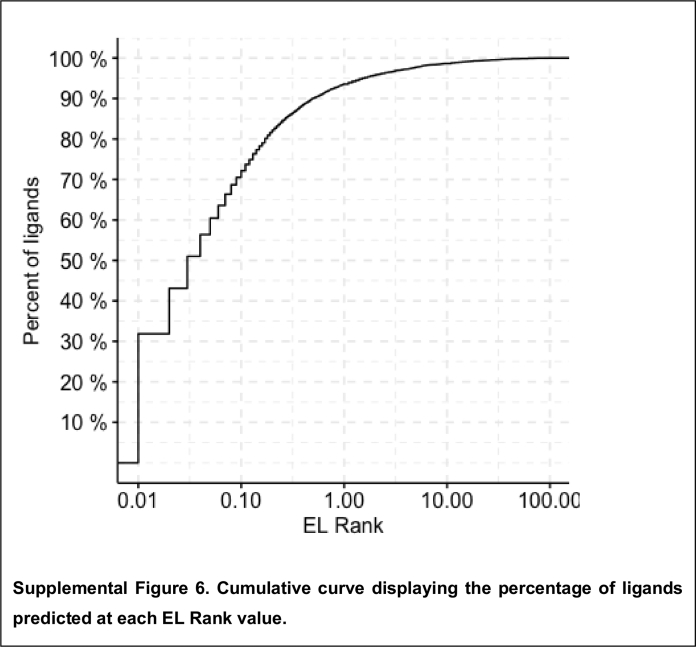


Next, we wanted to verify that the phenomenon was still detected using data derived from ELs. For each allele in Table 1, we determined thresholds for IC_50_ and EL rank that corresponded with 80% of ligands being predicted as binding (Figs. 3 and 4*A*, supplemental Table S1). This level of sensitivity is roughly equivalent to what is afforded by the commonly used 500 nM threshold (11) and is a reasonable level that captures the majority of epitopes while still providing adequate specificity. Cumulative plots showing the fraction of ligands at each IC_50_ and EL rank threshold are presented in supplemental Figs. S5 and S6.

**Fig. 5:**
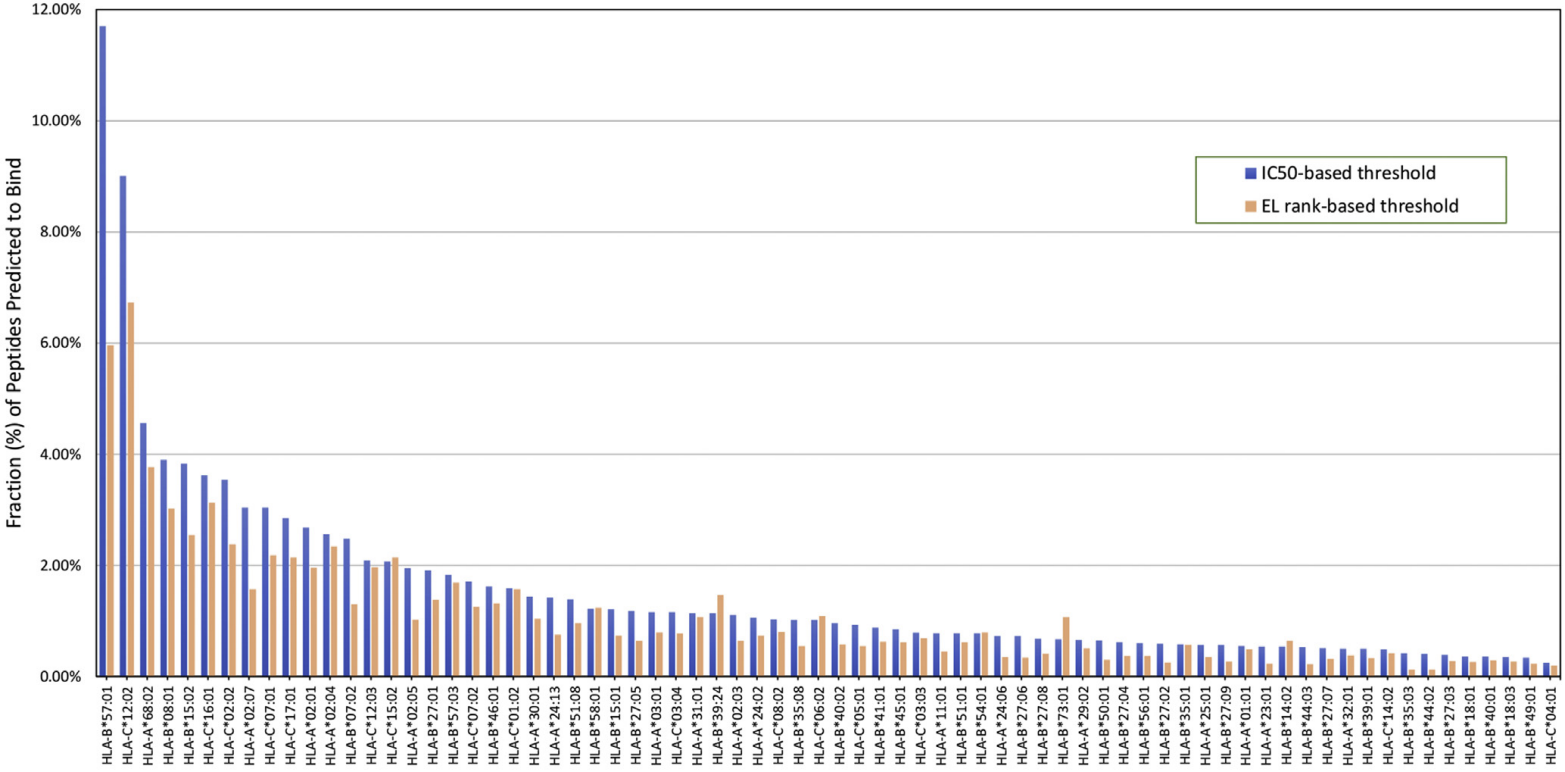
**The predicted binding repertoire varies by HLA class I allele.** The fraction of peptides predicted to bind using IC_50_ (*blue*) and EL rank (*orange*)–based allele-specific thresholds (sensitivity set to 80%) is shown. EL, eluted ligand; HLA, human leukocyte antigen.

**Supplemental_Figure-7:**
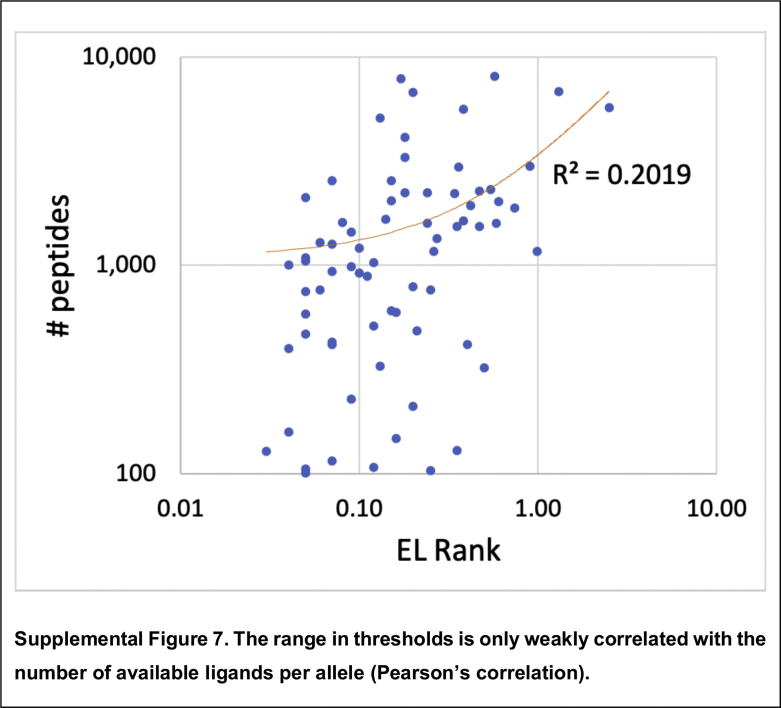


**Supplemental_Figure-8:**
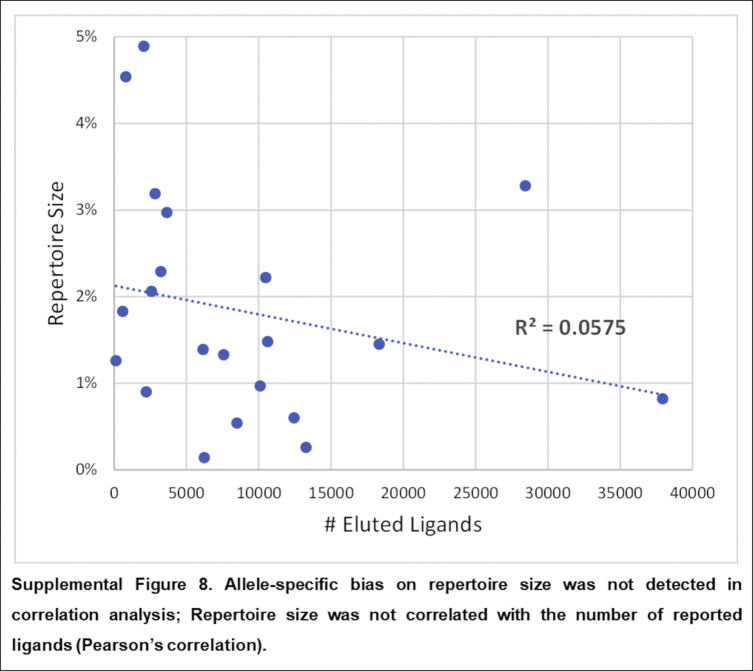


We found that the IC_50_ values corresponding to 80% of the ligands ranged over more than two logs from 22 nM up to 14,793 nM (mean 1459 nM; median 468 nM). Likewise, EL rank values ranged between 0.03 and 2.50 (~2 logs, mean 0.27; median 0.16, supplemental Table S1). Of note, the number of ligands available for each allele was only weakly correlated with the different allele-specific thresholds (R^2^ = 0.2, Pearson's correlation, supplemental Fig. S7). Using these updated allele-specific IC_50_ and EL rank thresholds, we calculated repertoire sizes for each allele and confirmed the variability of repertoire sizes among different alleles (Fig. 5). We did not see any obvious clustering of alleles by repertoire size according to their supertype or similarity in nucleotide sequences. Given that changing a single or just a few residues can change the shape of the binding groove, one cannot presume that highly similar alleles would necessarily cluster by repertoire size. We also examined whether there was any bias at the allele level by plotting the repertoire size as a function of the number of reported ligands on which the analysis was based and detected no significant correlation (R^2^ = 0.06, Pearson's correlation, supplemental Fig. S8).

These observations are important, as the ELs are not identified or selected based on predicted binding. Reproducing the analysis of different repertoire sizes with ELs excluded that the previous observations were due to biases in the predictive algorithms.

### Comparison of the Common IC_50_ and EL Rank Thresholds With Allele-Specific Thresholds for Predicting ELs in a Multiallele Setting

We next addressed whether it is preferable to use allele-specific thresholds or a common IC_50_ or EL rank threshold to maximize epitope prediction efficiency. We used the IC_50_ and EL rank thresholds corresponding to 80% sensitivity for each allele from Table 2 and computed specificity, PPV, NPV, and accuracy. To allow direct comparison with the common IC_50_ and EL rank thresholds, we identified the IC_50_ and EL rank values corresponding to 80% sensitivity in all ligands across all tested alleles (Fig. 3). We found that a set level of 80% sensitivity corresponded to an EL rank value of 0.17% and an IC_50_ of 780 nM (supplemental Table S1).

Using allele-specific thresholds provided similar overall specificity compared with the common EL rank threshold (Table 3a). Most importantly, however, the allele-specific thresholds by definition eliminated the variable performance of predictions that were observed when utilizing a common threshold. Indeed, when using the common EL rank threshold of 0.17%, the sensitivity on a *per allele* basis ranged from 46% to 100%, with an SD of 12.97 (Table 3a, supplemental Table S2). In contrast, in the case of allele-specific thresholds, the sensitivity on a *per allele* basis was by definition 80% with an SD of zero (Table 3a, supplemental Table S2). We obtained similar results when considering IC_50_ values (Table 3b and supplemental Table S2).

**Table 3:** Performance of allele-specific and common thresholds

Section	Threshold dataset	Forced sensitivity	Test dataset	Threshold type	Statistic	Sensitivity (%)	Specificity (%)	PPV	NPV	Accuracy (%)
a	Eluted ligands (EL rank)	80% in eluted ligands	Eluted ligands	Common	Mean	80.00	99.24	51.56	0.99	99.05
					SD	12.97	0.18	7.98	0.00	0.23
				Allele specific	Mean	80.00	98.89	52.00	0.99	98.71
					SD	0.00	1.19	19.32	0.01	1.18
b	Eluted ligands (IC_50_)	80% in eluted ligands	Eluted ligands	Common	Mean	80.00	98.22	41.46	0.98	98.04
					SD	19.11	1.57	19.29	0.02	1.46
				Allele specific	Mean	80.00	98.46	44.21	0.98	98.28
					SD	0.00	1.81	16.85	0.02	1.79
c	Eluted ligands (EL rank)	80% in eluted ligands	50 epitopes	Common	Mean	51.00	99.36	30.53	0.99	99.10
					SD	11.56	0.20	10.10	0.00	0.22
				Allele specific	Mean	58.40	98.96	27.90	0.99	98.75
					SD	12.75	0.82	11.02	0.01	0.79
d	Eluted ligands (EL rank)	80% in epitopes	50 epitopes	Common	Mean	80.00	96.99	12.93	0.97	96.90
					SD	9.80	0.82	3.52	0.01	0.81
				Allele specific	Mean	80.40	96.21	13.42	0.96	96.13
					SD	9.88	3.07	6.18	0.03	3.03
e	Eluted ligands (EL rank)	80% in epitopes	45 epitopes	Common	Mean	81.85	96.69	10.77	0.97	96.62
					SD	6.19	0.75	1.68	0.01	0.73
				Allele specific	Mean	84.07	95.34	11.53	0.95	95.29
					SD	11.19	3.80	7.04	0.04	3.75
f	Predicted binders	80% in epitopes	50 epitopes	Common	Mean	80.00	96.99	12.93	0.97	96.90
					SD	9.80	0.82	3.52	0.01	0.81
				Allele specific	Mean	80.00	96.53	15.52	0.97	96.44
					SD	9.80	1.97	13.66	0.02	1.94
g	Predicted binders	80% in epitopes	45 epitopes	Common	Mean	81.85	96.69	10.77	0.97	96.62
					SD	6.19	0.75	1.68	0.01	0.73
				Allele specific	Mean	84.07	95.89	9.78	0.96	95.83
					SD	9.37	1.76	2.85	0.02	1.72

We tested various common and allele-specific thresholds derived from different dataset and scaled to predict either 80% of ligands or 80% of T-cell epitopes. Threshold dataset: Peptide dataset from which thresholds were derived. Test dataset: Peptide dataset in which threshold performance was measured.

### Allele-Specific Thresholds Derived From ELs Perform Comparably to the Common Threshold in Predicting Actual T-Cell Epitopes

We next determined how the allele-specific EL rank thresholds that were established based on 80% sensitivity for each allele separately compared with the common EL rank threshold that was set based on 80% sensitivity for all alleles combined (0.17%). For this analysis, we used 50 randomly selected T-cell epitopes for each tested allele together with the random background control set. Accordingly, we used counts of TPs, TNs, FPs, and FNs to calculate performance for each threshold (supplemental Table S3).

First of all, we noted that the sensitivity provided by the EL-defined thresholds was not adequate to capture 80% of the T-cell epitopes; the sensitivities for common and allele-specific thresholds were only 51% and 58.4%, respectively (Table 3c, supplemental Table S3). Thus, the allele-specific threshold offered a limited gain in sensitivity. We next compared the SD of sensitivity for allele-specific and common thresholds to determine whether allele-specific thresholds would yield more consistent sensitivity values. We conducted this analysis as the SD expresses how much variation exists in the predictions going from one allele to the next. At the common EL rank threshold of 0.17%, the sensitivity for the 50 epitopes set on a *per allele* basis had an SD of 11.56, while the SD in the case of allele-specific thresholds was 12.75 (Table 3c, supplemental Table S3). Surprisingly, the variation in performance of allele-specific thresholds did not improve compared with common thresholds. The use of the allele-specific IC_50_ thresholds was also associated with similar results (data not shown).

To allow a more direct comparison, we adjusted the common threshold and allele-specific thresholds to an average 80% sensitivity. This was accomplished by increasing the common and allele-specific thresholds by a factor of 0.05 in a stepwise manner until an average sensitivity of 80% was achieved (Fig. 3*B*). As a result, common and allele-specific thresholds were increased by 6.5 and 4.6-fold, respectively, and performance was measured in the 50-epitope dataset (supplemental Tables S1 and S3). Similar to our observations when predicting ELs, the use of allele-specific thresholds provided similar overall specificity compared with the common threshold. The specificity for the 50 epitopes set when using the common threshold of 1.1% had an SD of 0.82, while the SD when using allele-specific thresholds was 3.07 (Table 3d, supplemental Table S3). Again, the variation in performance of allele-specific thresholds did not improve compared with common thresholds.

To validate these results, we assembled a second, independent set of epitopes. We chose not to reuse any of the peptides included in the 50 epitopes dataset. Several alleles had few or no remaining untested peptides beyond the 50, and we selected 45 peptides per allele from a subset of six alleles that still had sufficient untested peptides available to pick from. The performance on this independent dataset was comparable with what was observed with the 50-epitope datasets (Table 3e, supplemental Table S4). The EL rank thresholds that corresponded to 80% of T-cell epitopes being predicted are shown in Figure 4*B* and listed in supplemental Table S1.

### Allele-Specific Thresholds Derived From Binding Predictions Perform Comparably to the Common Threshold in Predicting T-Cell Epitopes

We next explored the performance of different thresholds based on BA predictions rather than ELs in predicting actual T-cell epitopes. To accomplish this, we used the common and allele-specific percent of background peptides predicted to bind at or below 500 nM (supplemental Fig. S3). These percentiles were increased in a stepwise manner by a factor of 0.1 until an average sensitivity of 80% was achieved in the 50-epitope dataset. As a result, the common and allele-specific thresholds were increased by 66.2 and 79.3-fold, respectively (Fig. 3*C*). We measured the performance of these thresholds in the 50-epitope dataset (Table 3f, supplemental Table S3). We also validated these results in the independent epitope datasets (Table 3g, supplemental Table S4). The EL rank thresholds based on binding predictions that corresponded with 80% of T-cell epitope being predicted are shown in Figure 4*C* and listed in supplemental Table S1.

These results show that the thresholds set based on binding predictions again perform overall similar to thresholds set based on eluted data. These results further indicated that overall, only a minor difference exists between using a common threshold *versus* allele-specific thresholds when the overall analysis is considered.

Finally, we also assessed the performance of the commonly used EL rank thresholds of 2% and 0.5% (supplemental Fig. S3). First, we evaluated the prediction of ELs. As expected, using a 2% threshold increased sensitivity; this was, however, at the expense of a dramatically decreased PPV and lower specificity and accuracy. Using a 0.5% threshold also increased sensitivity with a slight decrease in specificity and accuracy. There was, however, again a large decrease in the PPV. Given the low proportion of positives to negatives and the large number of ELs in an experiment, these thresholds would likely identify a few more TPs at the expense of many more FPs when compared with using our proposed common or allele-specific thresholds.

### Allele-Specific Thresholds Provide an Advantage for Selected Alleles

We found that overall, predicting epitopes using allele-specific thresholds performs comparably to what was observed when using a common threshold. Hence, when predictions for many different alleles are being performed, a common threshold might be practical, whereas if predictions are performed for a few selected alleles, allele-specific thresholds might be more advantageous.

To demonstrate this, we selected two alleles with very different allele-specific thresholds, namely HLA-A∗02:01 with an EL rank threshold of 2.62 and HLA-A∗11:01 with an EL rank threshold of 0.41 and combined the epitopes and background peptides for these two alleles. We then compared the performance of allele-specific thresholds to the common threshold of 1.11. We found that the precision is comparable when allele-specific or common thresholds are used; however, the sensitivity is considerably higher when allele-specific thresholds are used (supplemental Table S5).

## Discussion

In the present study, we investigated the performance of different strategies to perform predictions for multiple HLA class I alleles simultaneously. We found that strategies that use common thresholds generally applied to different alleles have similar overall performance compared with strategies utilizing allele-specific thresholds. This is true regardless of whether the thresholds were derived based on MHC-binding predictions or ELs. It would thus appear that if predictions for many different alleles are to be performed simultaneously, a common threshold might be more practical, whereas if predictions are performed for one or a few selected alleles, allele-specific thresholds are likely to allow for more consistent results. Here, we provide sets of thresholds associated with 80% sensitivity, which roughly corresponds to the original sensitivity level used to define the commonly used 500 nM threshold (supplemental Table S1).

One of the first issues we addressed was whether we could reproduce the finding by Paul *et al.* (6), highlighting that different alleles are associated with varying repertoire sizes, defined as the fraction of peptides predicted to bind in a control background set of peptides. Indeed, even with the most current NetMHCpan algorithm, the observation was reproduced and expanded to a larger number of alleles. As Paul *et al.* used binding data in their 2013 study, we next asked whether the findings could also be reproduced using an EL dataset. This dataset of ELs was generated by an entirely different method that does not involve any binding predictions, thus avoiding the danger of methodological self-referencing. The data clearly confirmed that different alleles are associated with different allele-specific thresholds to predict 80% of peptides. This is of significance, as it confirms the previous observations by Paul *et al.* (6), with a set of thresholds derived from a completely independent methodology.

Variability in the repertoire size among MHC alleles and the associated effects on the immune response against various diseases is a topic that is being actively researched. Correlations between repertoire sizes and cell-surface expression of MHC have been reported. Interestingly, several alleles with wide repertoires, that is, promiscuous alleles, were found to be associated with resistance to a variety of common diseases. In contrast, some alleles with narrow repertoires, that is, fastidious alleles, were reported to be associated with resistance to HIV progression (12, 13). One hypothesis that was suggested to explain this phenomenon is that during T-cell development in the thymus, promiscuous MHC molecules might present so many self-peptides that negative selection would deplete too many developing T-cells, which might be counteracted by reducing cell-surface expression of those alleles to reduce the extent of negative selection (14, 15). To explain these observations from an evolutionary view, it was suggested that promiscuous MHC alleles act as so-called “generalists” and fastidious alleles as “specialists.” Generalists are expected to provide sufficient protection against a wide variety of common pathogens but might fail to protect against new and virulent pathogens. In these cases, specialists might be particularly capable of presenting special peptides from the new pathogen (14, 15).

Another mechanism that reportedly has an effect on repertoire size is tapasin dependence. Tapasin is a critical component of the MHC class I peptide loading complex where it mediates the interaction between newly assembled MHC class I molecules and the transporter associated with antigen processing. Tapasin facilitates the loading of MHC with high-affinity peptides and promotes dissociation of low-affinity peptides (16). It was shown that some MHC class I alleles are tapasin dependent, whereas others are not (17, 18). Tapasin-dependent alleles require the peptide-loading complex to load peptides, and thus only high-affinity peptides are likely able to dissociate tapasin from the MHC peptide-binding groove. In contrast, tapasin-independent alleles do not require the peptide loading complex to load peptides, which enables the binding of peptides that may be of low affinity, resulting in a wider repertoire size (19). Bashirova *et al.* measured tapasin dependence levels of around 100 HLA alleles. We compared the top three tapasin-dependent HLA alleles Bashirova *et al.* reported, that is, B∗50:01, B∗44:03, and B∗44:02, and found that these alleles have narrow repertoire sizes in our analysis (Fig. 5). The top three alleles that our analysis identified as alleles with wide repertoires, that is, B∗57:01, C∗12:02, and A∗68:02, were reported as highly tapasin independent by Bashirova *et al.*

**Supplemental_Figure-9:**
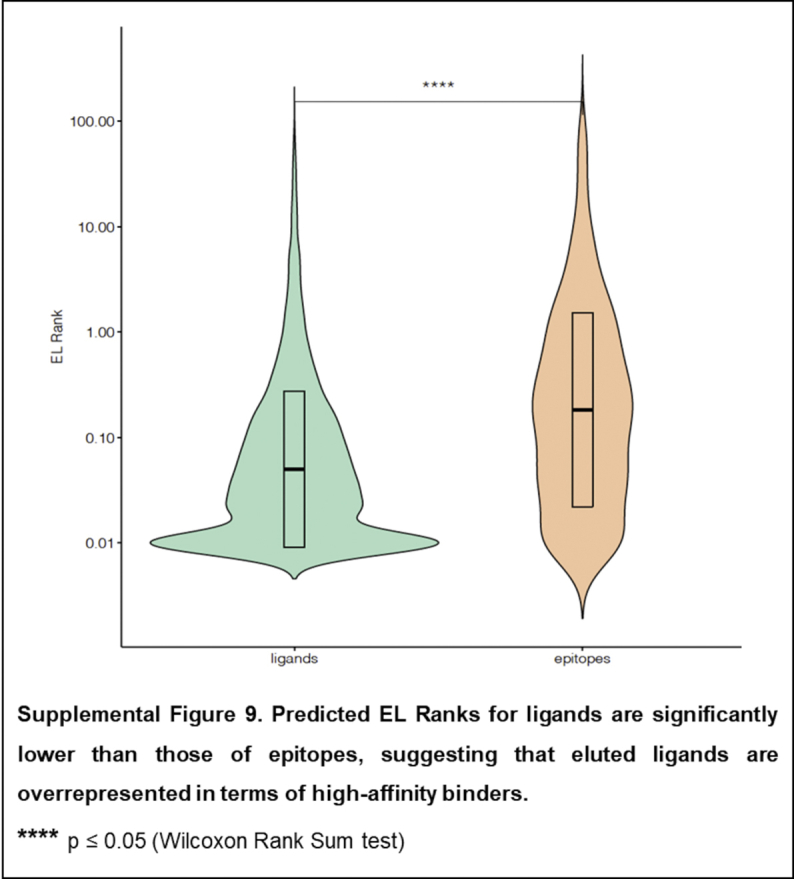

Ultimately, the purpose of bioinformatic predictions is, in most cases, not to predict ELs but rather to predict T-cell epitopes. To address this point, we curated sets of T-cell epitopes restricted by various HLA class I alleles and applied the thresholds defined with the eluted peptides to predict actual T-cell epitopes. We found that, to reach 80% sensitivity, the thresholds needed to be significantly adjusted. The thresholds associated with capturing 80% of the ELs only captured about 60% of the epitopes. The fact that a looser threshold needed to be used to predict epitopes rather than ELs is of practical importance and suggests that ELs are overrepresented in terms of high-affinity binders as compared with actual T-cell epitopes (supplemental Fig. S9). It is possible that lower affinity ligands might be lost during HLA purification, skewing the ligand affinity distribution. Alternatively, it is also possible that few copies of relatively lower affinity ligands might go undetected in the EL sequencing but be nevertheless sufficient to activate T-cells. Indeed, it has been shown that a few or even a single epitope copy per cell might be sufficient for T-cell activation (20, 21, 22).

Strikingly, we also found that the overall performance of allele-specific thresholds and common thresholds was equivalent when a relatively large number of alleles was considered. This likely reflects the fact that while performance varies over different alleles, the average performance is relatively stable and constant. In the case of allele-specific prediction, the fact that intrinsically vastly different number of peptides are predicted from one allele to another impacts overall performance and skews it toward over-representing the performance of alleles with larger repertoire sizes. Ultimately, for the sake of simplicity in global prediction, it is therefore preferable to use a “common threshold” strategy. But conversely, in the case of performing prediction for only one or few alleles, the use of allele-specific thresholds would be preferable. Because we make all the different thresholds available, each user can select a threshold strategy and methodology to fit their needs (supplemental Table S1).

We confirmed previous studies that EL rank–based predictions outperform IC_50_-based predictions (4). EL data contain important data such as sequence motifs associated with processing and allele-specific information about peptide length preferences. It was previously shown that including allele-specific length preferences in machine learning approaches improved predictions of ELs and epitopes (5, 23). The fact that these allele-specific length preferences are included in ligand elution data is a major advantage of utilizing training data based on ELs instead of BA.

One limitation of our study is that we only included 9-mer peptides. Future studies including various peptide lengths could provide more accurate insights into allele-specific thresholds and repertoire sizes. Another drawback is the limited number of epitopes available for analysis. Studies including a larger number of epitopes, given more epitopes become available in the future, could shed more light into our interesting finding that ELs are associated with significantly better prediction scores than epitopes.

## Data availability

T-cell epitope and eluted ligand source data analyzed during this study were obtained from the Immune Epitope Database (https://www.iedb.org/). Analyzed datasets generated during this study are included in this article and in the accompanying supplemental data.

## Supplemental data

This article contains supplemental data (6).

## Conflict of interest

The authors declare no competing interests.

## Figures and Tables

**Supplemental_Figure-1_1:**
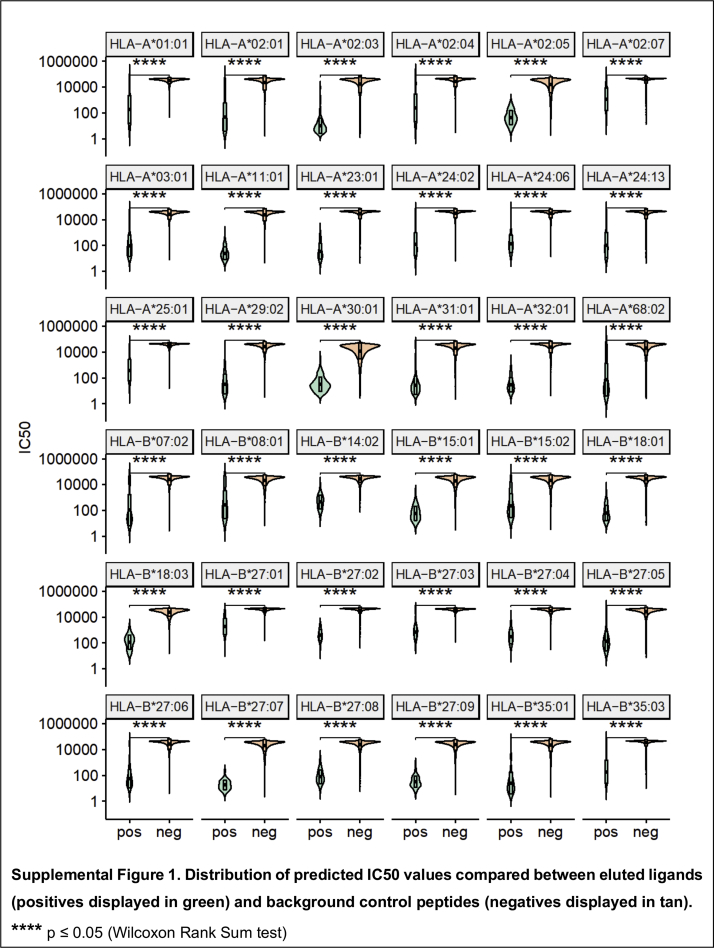


**Supplemental_Figure-1_2:**
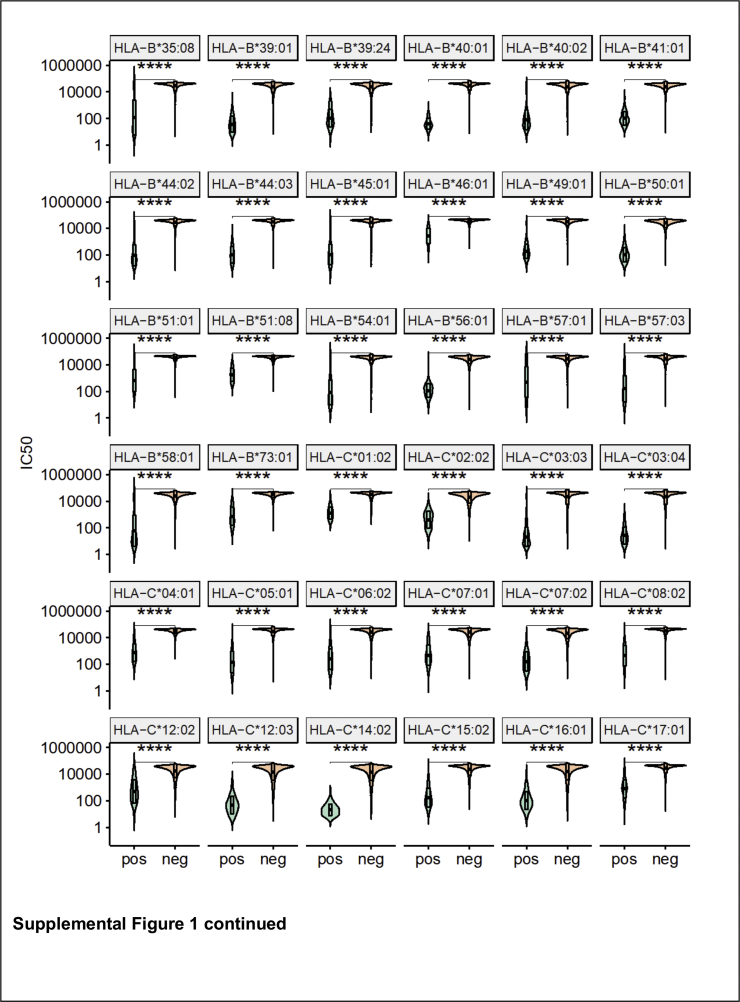


**Supplemental_Figure-2_1:**
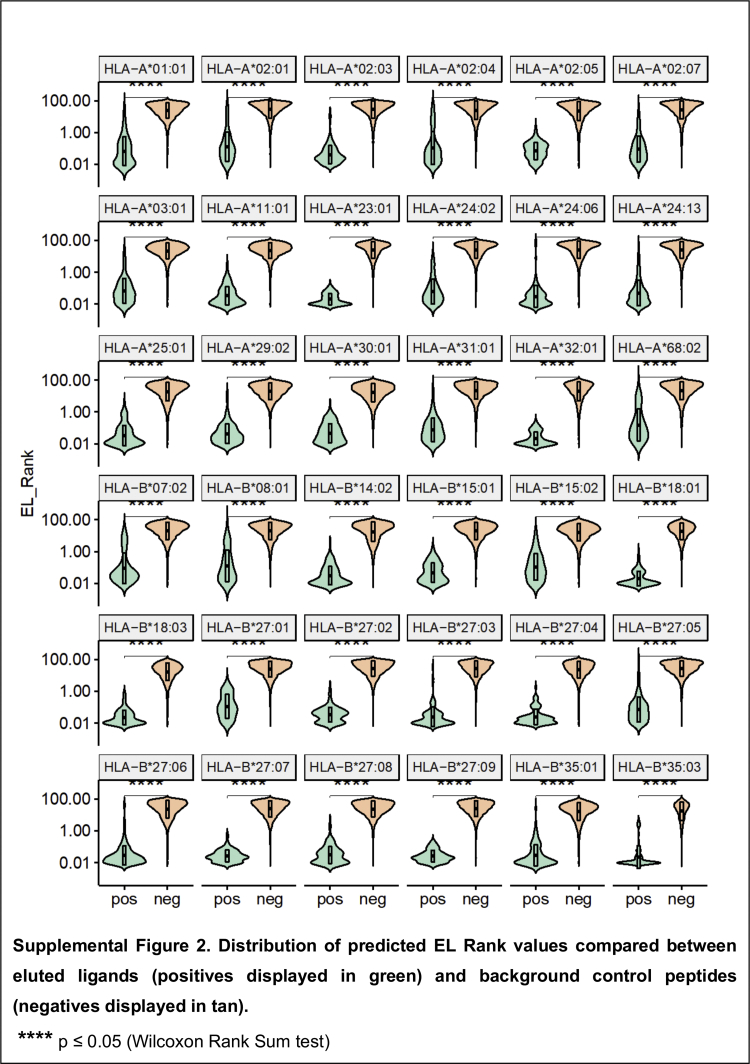


**Supplemental_Figure-2_2:**
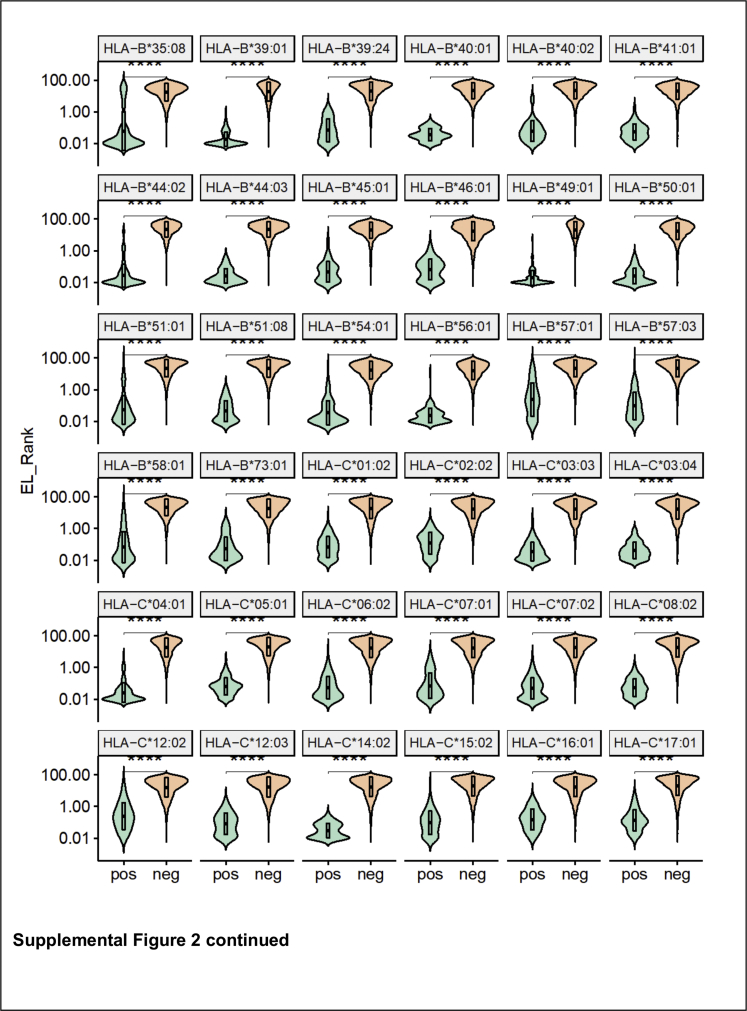

